# Being pregnant and becoming a parent during the COVID-19 pandemic: a longitudinal qualitative study with women in the Born in Bradford COVID-19 research study

**DOI:** 10.1186/s12884-023-05774-4

**Published:** 2023-07-04

**Authors:** Cath Jackson, June Brawner, Matthew Ball, Kirsty Crossley, Josie Dickerson, Nimarta Dharni, Diego Garcia Rodriguez, Ella Turner, Laura Sheard, Helen Smith

**Affiliations:** 1 Valid Research Ltd, Sandown House, Sandbeck Way, Wetherby, LS22 7DN UK; 2 Royal Society, London, UK; 3 Justice Studio, 10 Portfleet Place, De Beauvoir Road, London, N1 5SZ UK; 4 Bradford Institute for Health Research, Temple Bank House, Bradford Royal Infirmary, Duckworth Lane, Bradford, BD9 6RJ UK; 5 Institute of Applied Health Research, University of Birmingham, Birmingham, B15 2TT UK; 6 Faculty of Social Sciences, University of Nottingham, Nottingham, UK; 7 York Trials Unit, Department of Health Sciences, University of York, Heslington, YO10 5DD York UK; 8 Anthrologica, Oxford, UK

**Keywords:** Pregnancy, Maternity, Antenatal, Birth, Perinatal, Postnatal, Health visitor, Midwife, COVID-19, Pandemic

## Abstract

**Background:**

Uncertainty around the risk of COVID-19 to pregnant women and their babies prompted precautionary restrictions on their health and care during the pandemic. Maternity services had to adapt to changing Government guidance. Coupled with the imposition of national lockdowns in England and restrictions on daily activities, women’s experiences of pregnancy, childbirth and the postpartum period, and their access to services, changed rapidly. This study was designed to understand women’s experiences of pregnancy, labour and childbirth and caring for a baby during this time.

**Methods:**

This was an inductive longitudinal qualitative study, using in-depth interviews by telephone with women in Bradford, UK, at three timepoints during their maternity journey (18 women at timepoint one, 13 at timepoint two and 14 at timepoint three). Key topics explored were physical and mental wellbeing, experience of healthcare services, relationships with partners and general impact of the pandemic. Data were analysed using the Framework approach. A longitudinal synthesis identified over-arching themes.

**Results:**

Three longitudinal themes captured what was important to women: (1) women feared being alone at critical points in their maternity journey, (2) the pandemic created new norms for maternity services and women’s care, and (3) finding ways to navigate the COVID-19 pandemic in pregnancy and with a baby.

**Conclusions:**

Modifications to maternity services impacted significantly on women’s experiences. The findings have informed national and local decisions about how best to direct resources to reduce the impact of COVID-19 restrictions and the longer-term psychological impact on women during pregnancy and postnatally.

**Supplementary Information:**

The online version contains supplementary material available at 10.1186/s12884-023-05774-4.

## Background

The COVID-19 pandemic impacted on health and social life in every country globally. On 26 March 2020 the UK Government implemented a nationwide lockdown. Schools, non-essential shops and businesses closed, health and social care provision was reduced, and daily activities were restricted; in an attempt to limit the number of deaths, severe COVID-19 cases and pressures on the National Health Service (NHS) [1]. At the time, evidence on the clinical implications of COVID-19 for pregnant women and unborn babies was scarce and advice changed rapidly. Pregnant women were initially considered at moderate risk of severe illness and advised to ‘shield’ (remain at home unless seeking urgent medical care or medical attention for the birth of their baby, or fleeing danger) [2, 3]. When vaccination roll-out commenced, in December 2020, the UK’s Joint Committee on Vaccination and Immunisation (JCVI) initially cautioned against the COVID-19 vaccination for pregnant women due to lack of safety data [4]. This advice was reversed on 30 December 2020 when pregnant women were prioritised for vaccination [2, 3, 5].

Antenatal and postnatal care services had to adapt promptly to changing Government guidance. In October 2020, the Royal College of Midwives (RCM) and the Royal College of Obstetricians and Gynaecologists (RCOG) issued a statement recommending a minimum of six face-to-face antenatal consultations and a minimum of three postnatal contacts, with a preference for remote and consolidated appointments where possible. Telephone checks for COVID-19 symptoms were advised ahead of any face-to-face appointments [6]. Intrapartum services were also altered from March 2020, with low-risk women considered for non-hospital induction and labour; women monitored for risk and need before being admitted for caesarean sections; and restrictions on partner attendance in maternity services [6–8].

These modifications to maternity services led to substantial workforce changes, a reduction in appointments and the increased use of remote technology. The RGOG [9] found that 53% of junior doctors in obstetrics and gynaecology units’ junior staff were redeployed to other specialties, and at least one in five other staff members were unavailable for patient-facing clinical work in 40% of units at the peak of the pandemic. General and specialist maternity services reduced antenatal and postnatal appointments by 70% and 56% respectively during the pandemic, particularly for low-risk women, and 85% of sites reported using remote appointments, usually telephone, particularly in early pregnancy [7]. The substantial and rapid service changes also impacted on workforce wellbeing. In one survey almost 30% of respondents (*n* = 12,010) reported experiences indicative of post-traumatic stress disorder three-months after the first pandemic peak [10], and redeployment to other clinical areas without adequate clinical or infection control training was associated with adverse psychological effects [10].

Emerging evidence suggests these widespread service changes caused unintended negative consequences for pregnant women, including essential clinical care being overlooked, confusing advice, distress and emotional trauma [11]. Where home-based visits were replaced with virtual consultations, these appointments rarely met women’s needs, particularly for new-born care and guidance on infant wellbeing [12]. Women also found virtual consultations were impersonal, and as a result felt too embarrassed to discuss their mental health concerns [13]. Further to this, partner attendance and visiting were restricted in maternity units; a measure designed to keep women, babies and health workers safe. However, just weeks after restricting partner access to services there were reports of anxiety among women, feelings of isolation and lack of support [11]. Subsequent reports highlight the psychological distress pregnant women suffered due to disrupted birth plans and fear of perinatal COVID-19 infection [14–18]. All this evidence points towards maternity services that became more focused on the needs of the service and health workers, than providing ‘women-centred’ care that forefronts women’s unique needs, expectations and rights [19].

Much of the research conducted with pregnant women during the pandemic used surveys as a quick and easy way to engage and minimise contact between participant and researcher. Some researchers attempted interview-based research to obtain a more detailed understanding of women’s experiences [20–22]. Such studies found commonalities, including women’s feelings of isolation, lack of social support, increased anxiety and poorer mental wellbeing [23–26]. However, what is missing from the existing literature is an understanding of what was important to women during a maternity journey that was interrupted by various and fast-changing COVID-19 restrictions. Recently published work on this topic is predominately cross-sectional and quantitative [14, 15, 27, 28]. The existing qualitative research has tended to focus on the initial phase of lockdown restrictions, both nationally [13, 20–22] and internationally [18, 23–26]. In summary, most studies are at a static time point and interview women once about their maternity experiences. Lacking in the literature is exploratory qualitative research undertaken from a longitudinal perspective with women about their maternity journeys during the pandemic. In this paper, we sought a rich understanding derived through qualitative interviews over time to understand what women wanted or needed and what was important to them in their maternity journey during the pandemic.

## Methods

### Study design

We conducted an inductive longitudinal (three-wave) qualitative study, using in-depth interviews with pregnant women and women with new-born babies. The study was designed to understand women’s experiences at three critical timepoints: in pregnancy, during labour and after childbirth, and the 12 months postnatal period.

### Study setting

The study was conducted in Bradford, a large ethnically diverse city in the North of England, with an estimated population of 542,100 (the fifth largest local authority in England) [29]. During the coronavirus pandemic, Bradford experienced a relatively high number of COVID-19 cases compared with the rest of the UK [30]. High rates of COVID-19 in areas like Bradford were linked to greater deprivation, high population density and a higher-than-average number of multi-generational households [31].

### Sampling and recruitment

The Born in Bradford (BIB) cohort study, hosted by the NHS, is an internationally recognised research programme tracking in detail the lives of over 30,000 Bradfordians to find out what influences the health and wellbeing of families [32]. Women enrolled in two BiB cohorts (Born in Bradford’s Better Start (BiBBS) and BiB4All) were invited to participate in the BiB COVID-19 research study—a longitudinal mixed-methods study exploring the health, social, and economic impacts of the COVID-19 pandemic, from May 2020 onwards [33]. A purposive sample of women (identified by HS and LS) who had completed the survey between May and October 2020 were invited to participate in this qualitative study with the intention of achieving a mix of ethnicity, parity, and deprivation status. Women who did not speak English were oversampled, as we were particularly interested in their experiences. The BIB team sent 47 women a study information sheet and then followed-up by telephone 1–2 weeks later to ask if they would be interested in taking part. Those indicating interest gave verbal permission for their contact details to be passed to the researcher (JB, ND) who then phoned them and scheduled the first interview at their convenience.

### Data collection

Wave 1 interviews took place from May to November 2020, Wave 2 from February to May 2021 and Wave 3 from August to October 2021. Interview length was between 37 and 105 min. Given the restrictions on face-to-face contact, all interviews took place by telephone. JB conducted the interviews in English and ND conducted the interviews in Urdu. Verbal, informed consent was obtained from each participant. The interviews were digitally recorded and transcribed verbatim. Interviews in Urdu were translated and transcribed directly into English. Given this was a longitudinal study, the 18 women who took part in the first wave of interviews were re-contacted via a telephone call or text message by the same researcher to take part in a second and third interview.

### Interview questions

All interviews were conducted using a topic guide (see Additional File 1), with questions derived from recent literature on the topic and informed by the larger mixed-methods study that highlighted issues to be explored in detail [33]. However, the format was flexible to allow participants to voice what they considered important. Each wave of interviewing focused on women’s experiences at a discrete time point: wave 1 on pregnancy, labour and birth, wave 2 on the first six months post-birth and wave 3 on 6–12 months post-birth. At each wave the key topics explored were physical health and mental wellbeing; access to and use of services; relationships with partner and family; and general impact of the COVID-19 pandemic on these experiences.

### Analysis

Framework analysis was undertaken in five steps [34]. For each wave of interviews a sub-group of the research team (JB, MB, DG, CJ, ET) independently read a third of transcripts to develop the coding framework (see example in Additional File 2). Categories reflected some of the broader topics covered in the topic guides. Codes were developed from careful review of participants’ responses whilst sub-codes reflected more nuanced responses. The codes/sub-codes from the frameworks were then systematically applied to all transcripts using Dedoose software. The coded data were exported into Word documents as summarised data for review and subsequent regrouping into themes. Key themes and patterns within these themes were identified and differences in responses between ethnicity and parity explored. Preliminary findings were discussed with the full research team after which the themes were finalised and written up. The analysis was entirely inductive and we did not structure it on any existing theoretical frameworks. The final step was a longitudinal synthesis. The data from each wave were synthesised (by CJ, HS, LS) to identify over-arching themes that captured women’s views and experiences of pregnancy, childbirth, and having a new baby during 18 months of a global pandemic.

### Context of the COVID-19 pandemic during the study period

Wave 1 interviews, and the months prior on which women reflected, (February to November 2020) took place during the first nine months of the pandemic. During this time the first national lockdown was introduced (26 March 2020) with pregnant women advised to “shield” at home until the end of May. Gradual opening of schools and shops, as well as lifting of social contact restrictions commenced from June. In mid-October a three-tier alert system was introduced whereby different areas were allocated to a tier (set of restrictions) according to their COVID-19 situation; Bradford entered tier 3 (very high alert) [35]. The national modifications to antenatal and postnatal services [1, 2, 6, 9, 36] were in place in Bradford. These included closure of homebirth services until end June 2020, and partners only being allowed for 12-week scans and to attend during active labour until end September 2020. The second national lockdown commenced on 5 November 2020 [35].

Wave 2 interviews, and the months prior (September 2020 to May 2021) were carried out at the end of the second lockdown (2 December 2020) and return to the tier alert system. Restrictions were relaxed in December 2020 for most of England, with Bradford re-entering Tier 3 at the end of December. The third national lockdown commenced 6 January 2021. The “roadmap for lifting lockdown” occurred in March to May, when there was a gradual opening of society and restrictions for social contact were lessened [35]. During this time partners could attend all stages of labour (from end September 2020) and all antenatal scans from end January 2021.

In Wave 3 and the months prior (March to October 2021) legal limits on social contact ended and the final closed sectors of the economy re-opened (July to September 2021) [35]. The maternity services protocols in place at the end of Wave 2 remained.

## Findings

### Participant characteristics

Eighteen women were interviewed in wave one, 13 in wave two and 14 in wave three (see Table 1). Of the 18 women recruited at wave one, eight were White British, nine were of Pakistani heritage and one was White European. Two thirds had been born in Bradford. Two women of Pakistani heritage were interviewed in Urdu. Nearly two thirds had older children, most commonly one child. Nearly two thirds had professional or office jobs. The average age of women at first interview was 31.5 years.

**Table 1:** Participant characteristics

ID	Interviews completed	Age at first interview (years)	Ethnicity	First child/has other children	Number of other children	Born in Bradford?	Employment	Partner’s employment
W01	1, 2, 3	27	White European	First child	N/A	No	Retail	Transport
W02	1, 2, 3	35	Pakistani Heritage	First child	N/A	No	Business	Hospitality
W03	1, 2, 3	28	White British	Other children	One	Yes	Administration	Health and fitness
W04	1, 2, 3	39	White British	Other children	One	No	Education	Administration
W05	1	30	Pakistani Heritage	First child	N/A	Yes	NHS	Education
W06	1,2,3	36	Pakistani Heritage	Other children	One	Yes	Administration	NHS
W07	1	28	White British	First child	N/A	Yes	Financial services	Private sector
W08	1, 2, 3	32	White British	First child	N/A	Yes	Public sector	Private sector
W09	1, 3	28	Pakistani Heritage	Other children	Two	No	N/A	Retail
W10	1, 2	33	White British	Other children	One	Yes	Health and fitness	Public sector
W11	1, 2, 3	39	White British	Other children	Three	Yes	Public sector	Private sector
W12	1, 2, 3	31	Pakistani Heritage	Other children	Two	Yes	Health and fitness	Transport
W13	1, 2, 3	28	White British	Other children	One	Yes	Education	Transport
W14	1	32	White British	Other children	Three	Yes	N/A	Education
W15	1, 2, 3	25	Pakistani Heritage	First child	N/A	Yes	Education	Civil service
W16^a^	1, 3	34	Pakistani Heritage	Other children	One	No	N/A	Hospitality
W17^a^	1, 2, 3	30	Pakistani Heritage	Other children	Three	No	N/A	Retail
W18	1^b^, 2, 3	26	Pakistani Heritage	First child	N/A	Yes	Public sector	NHS

^a^Interviews conducted in Urdu

^b^No transcript

We describe below what was important to women in their maternity journey during the pandemic, in their own words, using verbatim quotes to illustrate key points. Three aspects were identified as particularly important: women feared being alone at critical points in their maternity journey; the pandemic created new norms for maternity services and women’s care; and finding ways to navigate the COVID-19 pandemic in pregnancy and with a new baby. Each theme (and sub-theme) includes data from across the three waves (see Table 2), and where there are differences in perspectives by ethnicity or parity these are described.

**Table 2:** Outline of themes and sub-themesThemes

	Sub-themes
Theme 1: Women feared being alone at critical points in their maternity journey	Fear of being alone was felt most acutely during pregnancy
	Having a partner present during labour and childbirth made an important difference to women
	Aspects of women-centred care were compromised
	A lack of consistent “official” information intensified emotions associated with being alone
	Pregnant women felt their mental health deteriorated early in the pandemic
Theme 2: The pandemic created new norms for maternity services and women’s care	Perceptions of reconfigured services
	Women bore the brunt of rapid changes to postnatal care and community-based services
	Frustration with GP appointment booking and seeking alternatives
Theme 3: Finding ways to navigate the COVID-19 pandemic in pregnancy and with a new baby	Adapting to being labelled “at risk” during pregnancy
	Navigating birth preparation decisions during the pandemic
	Navigating social interaction for mental wellbeing

### Theme 1: women feared being alone at critical points in their maternity journey

This theme represents the persistent underlying narrative that women feared “*being alone*” during their pregnancy journey and at critical points in their routine care. The fear of not being with their partner was felt most acutely during pregnancy and when attending antenatal care. Women’s accounts also highlighted how aspects of women-centred care were often compromised, for example continuity of carer and effective information and communication.

#### Fear of being alone was felt most acutely during pregnancy

For most women not having a partner with them at routine 12 and 20-week scans made a difference to how they felt and coped. Women described their feelings about being alone for these appointments as “*terrifying*”, “*scary*”, “*daunting*”, “*freaked out*” and “*horrendous*”. They mainly worried about receiving bad news alone or having to break bad news to their partner. Women were also mindful of their partner “*being pushed out*”, which was manifest in not hearing the baby’s heartbeat, not being present to learn the baby’s sex and being unable to ask their own questions.

*It was hard because I couldn’t have my husband coming with me, like for the appointments, so I wanted to share it with him, but I couldn’t because he wasn’t allowed in. He couldn’t see the gender reveal scan and he couldn’t come to any of the antenatal appointments. And if I had like a worry, say if I couldn’t feel his* [the baby’s] *heartbeat, and I had to go in there on my own, like all scared and stuff. No-one there, for me it was a bit, yeah, daunting, like not a nice experience*.

(W15, Pakistani Heritage, First-time mother, Wave 1)

#### Having a partner present during labour and childbirth made an important difference to women

A common worry expressed by women was that they would have to experience labour and childbirth alone. Many described how they had attended the assessment and birth centres alone, with their partners allowed to accompany them once, during active labour. Some recalled partners waiting outside the facility in their cars to be called in. A few women had spent long periods of time (e.g. six hours, 24 h, overnight) during labour on their own.

*During my induction, my husband wasn’t allowed to be there, and I wasn’t happy with that because I would have liked him to be there for support. So he was only able to come down after they had taken me down to the labour ward to break my waters. I was alone a whole day. I felt quite, sort of, you know, anxious and I just felt as though I didn’t have any support with me.*

(W05, Pakistani Heritage, First-time mother, Wave 1)

In contrast, women whose partners had been with them throughout labour and birth recounted more positive experiences. One woman described herself as “*lucky*” because the hospital restrictions had changed a few weeks before, and another was relieved that her partner could be with her commenting that “*If he wasn’t there, I don’t think it would have been as positive as it was*”.

Most women said they accepted restrictions that meant their partners could only stay for a few hours after the birth, although some felt *“disappointed”* and *“upset”* that their partners were unable to support them. In two exceptional cases, partners had stayed for a whole day after the birth, and these women were *“grateful”* for their support.

#### Aspects of women-centred care were compromised

Women had a strong preference for face-to-face appointments, especially during pregnancy, which gave them *“peace of mind”*, left them reassured and confident that they had had a “*proper check-up”.* Some described feeling “*pushed out*” and “*unsupported*” by telephone appointments, typically commenting on the lack of physical examinations. Irrespective of whether this was a woman’s first or subsequent pregnancy, all believed that only having phone appointments would likely be hardest for those in their first pregnancy. Similarly, whilst women described online antenatal classes as “*informative*” and “*enjoyable*”, they missed being supported in person with practical hands-on experience, having opportunities to discuss issues with the midwife and to share experiences with other new parents.

*I’ve had a very easy pregnancy, I’ve not had no problems so I’ve felt OK, but I just feel like I don’t see how a midwife can see if you’re OK over the phone. They can’t. They can’t check your urine, they can’t check your blood pressure, they can’t check anything, all they can do is say, ‘are you okay’?* (W03, White British, Mother with other children, Wave 1)

*We did like an antenatal course but it was all just via Zoom so it wasn’t, it didn’t feel as maybe hands-on and practical as it could have been but obviously that’s just the way it had to be. But if you were there meeting the other couples and the lady running the course it could have been different. The face-to-face contact was missing.* (W02, Pakistani Heritage, First-time mother, Wave 2)

Women clearly valued continuity of carer with the same midwife, but most had seen several different midwives during their pregnancy. Consequently, they felt they had not developed a relationship with “their” midwife. Those who attended appointments via GP practices were more likely to see the same midwife consistently, and for these women the benefit was clear.

*Yeah, I didn’t have anyone familiar and that was a bit upsetting. I thought I’d have someone, and I’ll be happy but to be honest with you one went on a holiday, one’s pregnant, she’s working from home. I don’t know whether, maybe some of them were not working on that day if you know what I’m saying.* (W09, Pakistani Heritage, Mother with other children, Wave 1)

*I was lucky with her too because she’s been my midwife all the way through my pregnancy and then on the day that I go into labour, well yeah, she’s there and she was the one that delivered my baby. I couldn’t believe it because, you know, I know her and she kind of knows me. Because obviously you get, over the nine months you get to kind of know each other and it was like, and it just, as soon as I saw her face it was like all my anxieties disappeared.*

(W06, Pakistani Heritage, Mother with other children, Wave 1)

#### A lack of consistent “official” information intensified emotions associated with being alone

Government information about COVID-19, especially how it affected pregnant women, was said to be insufficient, unclear and constantly changing. This further confirmed women’s perceptions of being unsupported and intensified their worry and anxiety. Whilst acknowledging that everyone (including health workers) was “*in the dark*” with this new situation, women found midwives’ apparent lack of knowledge about how COVID-19 affects pregnant women and their unborn/new-born babies stressful.

#### Pregnant women felt their mental health deteriorated early in the pandemic

Two-thirds of women reported they had experienced low mood and/or mild anxiety during their pregnancy. Some related this to *“being in the house all the time”*, while for others, the above-described fear and experience of being alone, isolated from family and friends, unsupported by their partner and midwife had contributed to their low mood or anxiety.

*Oh, it’s been awful. Before where if I felt my anxiety was going a bit higher I would go out for a drive, I’d take the kids out, we’d go to a park, we’d just let loose, breathe in some fresh air, you know, clean oxygen and stuff like that, it was a different environment. But now you’re 24/7 stuck in the house.* (W12, Pakistani Heritage, Mother with other children, Wave 1)

### Theme 2: the pandemic created new norms for maternity services and women’s care

The substantial and rapid changes to maternity services during the COVID-19 pandemic inevitably affected women’s experiences. Women described the elements of care that were important to them, as well as the service changes that negatively impacted on the care they received.

#### Perceptions of reconfigured services

Overall, most women were positive about the care received in antenatal services, at the time of birth and immediately after. The consensus was that antenatal care services had adapted well and continued to provide a professional service, and that staff were *“doing their best”*.

*I think they’ve been brilliant, you wouldn’t know they’ve been affected, even though you know they have, even though I know like the midwives last week said they’ve got like loads of staff off, and she went, yeah, I’ve just pulled a 24-hour shift. And just still being professional through it all, and yeah, they’re just brilliant.* (W04, White British, First-time mother, Wave 1)

That said, some felt frustrated with the constantly shifting rules and hospital restrictions that impacted on their birth plans. Several of the women who were planning a home birth or pool birth in hospital explained how the decision was not made until the very last minute. Whilst in the end, almost all had the birth they had planned, some frustration with the process was evident.

*The rules of the midwives coming into the home were very strict, so they were obviously wearing masks and other PPE which I again was uncomfortable about because I thought everything I wanted about a home birth is being changed. It feels suddenly a very clinical environment, and they said that my children weren’t allowed to be there, which again just felt like… Yeah. All my choices were being taken away. I felt like we just had lots of battles to deal with and things to kind of confront and it didn’t feel like that should be the case when you’re having a baby.* (W04, White British, Mother with other children, Wave 2)

For one woman the constantly shifting rules and hospital restrictions left her feeling like she had no control over her pregnancy and birthing plan.

*I was very teary, very, very teary. I had panic attacks. I’d never had a panic attack before. I think it was the restrictions placed on us. I felt out of control and I felt panicked about what was going to happen. I felt like we’re going into the complete unknown with this baby compared to the other babies and I didn’t know how my maternity leave was going to go. I didn’t know how life was going to be, but just the waiting to hear on the news what I was allowed and not allowed to do, I think that had a bigger impact than I imagined it would do*.

(W11, White British, Mother with other children, Wave 1)

Later in the pandemic, when women had given birth, they tended to acknowledge the impact of the pandemic on postnatal care and women recognised the challenges faced by the NHS.

*I'd say they're doing like really well just to say that they've literally been thrown into it. To organise appointments and things like that, they really have done like the best that they can to ensure like the safety... I feel quite happy about the situation really. To say like it's been really tough on everybody, like the way the healthcare services have adapted.*

(W13, White British, Mother with other children, Wave 2)

Women mostly considered themselves and their new-born babies safe from COVID-19 and expressed confidence in hospital procedures; two thirds considered themselves and their new-born to be safe in hospital, trusting COVID-19 protocols. The other third was nervous about the risk of coronavirus infection and the impact of COVID-19 restrictions.

*I thought it was really safe. I’d already been* [in hospital] *in the summer when I’d got a kidney infection, and I knew that they were testing routinely for COVID. I knew I’d not been in contact with anyone with COVID. Erm… every time I’d been in for an appointment, I was wearing a mask, everyone was like wearing masks and being really safe. Obviously, I was nowhere near a COVID ward because the maternity unit is completely separate from everything else, so, actually, it was probably safer.* (W04, White British, First-time mother, Wave 2)

*I was nervous about it. You know even in the corridor when I was passing somebody or even when I had the baby, you know, people were coming to check on her, you know, they had PPE (personal protective equipment) on, everything like that. I just felt conscious about everything, with the whole COVID thing, I didn’t want to be in hospital longer than I needed to.* (W06, White British, Mother with other children, Wave 2)

#### Women bore the brunt of rapid changes to postnatal care and community-based services

Women generally had positive interactions with midwives and health visitors in the first eight weeks after birth, and most accepted that seeing a different midwife in the community as *“the norm”* now. Two women felt more anxious about receiving care from a *“new”* midwife and believed that information around new-born care conflicted with what had been imparted by midwives in hospital.

*I were a bit disappointed afterwards because it were a different midwife that I were seeing every time they’d come, it were never the same one and they were, sort of, telling me something different every time which, because obviously there were a different person. So it were just confusing and it didn’t make it any easier on the situation, whereas if it would have been one person and they were telling me the same thing every time I could have stuck to it or whatever.* (W10, White British, Mother with other children, Wave 2)

Once health visitors took over from midwives, up to half of the women felt that health visitors were less present than midwives had been. There several reasons for this. First, a lack of face-to-face contact for the routine 6–8 week appointment with health visitors was problematic for some women who felt physical checks of the baby and mother were important, and they valued the opportunity to discuss more “*intimate*” matters in the first few weeks after giving birth. However, there did appear to be more continuity of carer with health visitors, which allowed women to build a stronger and more familiar relationship with the same health visitor and was considered important for good quality care.

Second, women described a perceived lack of support between the 6–8 week and 6–9-month postnatal contacts. Many women wanted more contact between these scheduled visits and missed the reassurance that health visitors provide. New mothers particularly missed having feedback that their baby was developing satisfactorily, and that they were “*doing a good job*”. They also wanted advice on feeding, weaning and sleeping.

*Then the health visitor came, she gave me all the stuff for six months, and said, “I probably won’t see you again”, and I’ve not heard from her. Which is really scary. I worry about all those mothers that are, that need a bit of support or, and they haven’t got a health visitor checking in, not even a phone call until six months.* (W04, White British, First-time mother, Wave 2)

That said, half the women, predominantly of Pakistani Heritage, had contacted or tried to contact the health visitor team between scheduled visits. Most had been given a number to use and were satisfied with the support they had received. Two other women, both with older children, appreciated having longer pre-scheduled conversations with a health visitor, and telephone breastfeeding support respectively.

The third reason related to the absence of health visitor-led clinics. Several women mentioned wanting to have their baby weighed, with a few recalling the drop-in clinics they had attended with previous children. Some women reported seeking informal support and information from other sources including Google, Facebook groups for parents, friends and family members with children and a few weighed the baby themselves for “*peace of mind*”. Women who mentioned this self-help approach generally said they would have preferred professional input.

*There have been no calls to make sure of anything which, which yeah you are then left on your own. Nobody really discussed weaning, how to wean your kid onto solids. I would have appreciated more support in the first year, just to check him up in-between, make sure he's developing okay because yeah, I know what he's like as a child but I don’t know what's happening inside his body. I don't know if everything's normal.* (W02, Pakistani Heritage, First-time mother, Wave 2)

Women also described positive experiences with health visitors. A common view was that accessing infant vaccinations was “*pretty straightforward*”; and face-to-face visits by the health visitors at 6–9 months were also well received, providing the reassurance the mothers had been seeking. Women typically saw a different health visitor to the one they had seen for the earlier visits, but many seemed to accept this as “*the norm*”.

*I’m a confident mum and I know kind of that he’s healthy and thriving I just wanted somebody else to check him over, so she* [the health visitor] *did come, and she was fine with it. She didn’t stay very long but she confirmed everything I thought that he was, you know, healthy and developing really well.* (W11, White British, Mother with other children, Wave 3)

#### Frustration with GP appointment booking and seeking alternatives

In the last wave of interviews, when most COVID-19 restrictions had lifted, women expressed frustration with accessing GP services. Women, mainly White British women with more than one child, complained about the booking process; they were weary of the time it took to get through on the telephone to speak with a receptionist. Some found alternatives to the GP, for example seeking pharmacist advice on whether a baby had chicken pox, asking a homoeopath to treat a baby’s rash or phoning 111. Whilst women we spoke to seemed to accept the inevitability of remote consultations, several complained that their requests to see a doctor face-to-face with their baby had been declined. Women wanted their judgement, as mothers, to be trusted about when their child should be seen by a doctor in-person.

*I don’t mind the telephone calls and the video calls for other things because it's actually quicker and more effective when I don’t actually need to see someone every time, but it would be useful if they trust kind of parent’s judgement as to when we do need to be seen.*

(W11, White British, Mother with other children, Wave 3)

### Theme 3: finding ways to navigate the COVID-19 pandemic in pregnancy and with a new baby

At each timepoint women revealed numerous examples of having to find their way, or steer themselves through, new or unknown circumstances associated with the pandemic. In this theme we identify a continuum of ‘navigating the pandemic’ from adapting to being labelled *“at risk”* during pregnancy, to navigating birth preparation decisions, and finally women steering themselves through the COVID-19 recovery period with a new baby.

#### Adapting to being labelled “at risk” during pregnancy

Many women recalled feeling anxious early in the pandemic when pregnant women were labelled *“at risk”* and required to shield at home. This was fuelled by an absence of information about the risks to themselves and their unborn babies, some panic amongst family members and *"a sort of paranoia”* about performing thorough preventive measures to protect themselves. When shielding at home in pregnancy, for some, their personal relationships became stronger, and they valued the opportunity to spend more time as a family. However, feelings of isolation were common and the inability to change routine or take a break from family was a challenge for some. Many women anticipated pregnancy being an exciting time but had not experienced this, and comparisons with previous pregnancies were generally negative. One woman described going through her pregnancy “*in hiding”*. Whilst most had kept in touch with close family and friends via telephone and video calls, they missed the impromptu advice and unspoken support afforded by in-person meetings.

*It was quite scary, to be honest, because obviously I was on the shielding list, and I didn’t go out anywhere. And there was just such a big sort of scare about the whole virus that you were just…reluctant to do anything, to go anywhere.*

(W05, Pakistani Heritage, First-time mother, Wave 1)

*If anything, we’ve kind of enjoyed it, like having that time together. Because usually we’re kind of like, you know, he’s at work, I’m at work, we come back, we eat, we sleep, that kind of thing, but because both of us have been working at home and we’ve had a lot more time together than we would normally have.* (W06, Pakistani Heritage, Mother with other children, Wave 1)

#### Navigating birth preparation decisions during the pandemic

It was clear that many women, especially those with other children, were making different decisions during pregnancy to those they would have made outside of a pandemic. Examples included partners living away for periods of time to ensure the woman’s safety leaving one feeling like a “*single mum”*, and conversely family members moving in to be available for childcare when the woman went into labour (otherwise not permitted during the first lockdown). Some felt uncomfortable that they might need to *“break the rules”* but could see no alternative.

*That’s probably hardest thing because you get fined for this and you get fined for that. I understand why they put them measures into place but if my husband can’t get home in time* [when I go into labour]*, who do I have here with me to help support me when no-one’s allowed in my house?* (W10, White British, Mother with other children, Wave 2)

A few cited the pandemic as the reason for choosing a home birth. Having the baby at home felt safer, more relaxed in terms of social distancing measures, easier in terms of childcare and their partner could be there throughout. There were also examples of women making decisions to avoid *“being alone”* in hospital. Examples included staying at home longer when having contractions and leaving hospital soon after the baby was born despite wanting to stay.

*I wasn’t happy. I felt sort of, you know, anxious and I just felt as though I didn’t have any support with me* [for a whole day after being induced]*. Now, because it was my first baby, ideally, I would have liked to have stayed in hospital to get a bit more support, a bit more advice. But because my partner wasn’t able to stay, nobody was able to come visit, I opted to go home. So, I gave birth and literally a few hours later, I went home because I wasn’t willing to just stay on my own.*

(W05, Pakistani Heritage, First-time mother, Wave 2)

#### Navigating social interaction for mental wellbeing

Descriptions of the early months with a new baby included *“hard”*, “*quite lonely”, “draining and isolating”* and *“claustrophobic”.* Many women reflected on the stress and low mood associated with feeling exhausted from caring for a new-born alongside feelings of isolation. They typically relied on their partner or their wider (mostly remote) network of family and friends for *“off-loading”* and support. WhatsApp and Facebook groups enabled them to share and receive advice and ask questions to other parents.

*You’re just trapped with a baby, you know, you’re just, it’s just you and a baby and suddenly you’ve got this thing to look after, and you’ve got no one else to bounce off or just whinge at or, who’s going through it the same time as you.* (W04, White British, Mother with other children, Wave 2)

*Now that we can’t meet, we speak only on WhatsApp. We post pictures, and I started giving him food and the one who is two weeks less than us, her, she started to give food to the baby, so we post what we give, and we speak on WhatsApp, and things like that, so we still keep in touch and give advice.* (W01, White European, First-time mother, Wave 2)

During the interviews in summer 2021, women were noticeably more positive in mood and expressed better mental health. Much of this change was attributed to easing of restrictions, being able to see friends and family, access informal support and plan holidays. They talked about attending social activities such as mother and baby groups, swimming pools and indoor soft play centres. Just a few felt “*wary”* or *“scared”* of crowded places and exposing their baby to new germs, although in general these feelings were subsiding over time.

*I think I’ve started to feel better about everything actually, I was feeling a bit doom and gloom before because it was a bit like oh, what, I don’t have anything to look forward to and you know, what’s going on and I want to go away and I want to do this and I want to do that. I think because there is going to be some changes coming up for me and I am going to kind of be getting out the house, there is going to be you know, something different.*

(W06, Pakistani Heritage, Mother with other children, Wave 3)

The importance of having somewhere to go with a new baby was clear. Many were mindful of the impact social restrictions had had on their baby’s social development, describing them as *“clingy”* and worrying that the baby did not know how to play with others. Most were pleased to now have an opportunity to expose the baby to other people, some observing that their babies were *“happy”* and *“excited”* to be around other children. Few opportunities to make friends with other parents having a baby at the same time was a recurring topic from the first interviews. However, by the final interview women began talking about enjoying chatting with other mums and arranging to meet up again.

*It’s been a lot better to be honest with you because I found that I’ve been able to get him into more classes for more interaction with other people, more interactions with babies, being able to take him to play groups and like swimming, you know, just to give him a bit more experience because obviously for the first six months he didn’t have anything other than either going for a walk.*

(W08, White British, First-time mother, Wave 3)

## Discussion

This longitudinal qualitative study has captured women’s candid accounts of what was important to them in their maternity journey during the pandemic. Three aspects were identified as particularly important: women feared being alone at critical points in their maternity journey; the pandemic created new norms for maternity services and women’s care; and finding ways to navigate the COVID-19 pandemic in pregnancy and with a new baby. A central thread through the three waves of the study was that support (in various forms) was important to women yet because of modifications made to maternity services during the pandemic women often lacked access to the support they needed. We found very few differences in views by women’s parity or ethnicity.

The persistent underlying narrative of women feeling alone at critical points in their care is concerning. Women reported attending routine antenatal scans alone, going to assessment and birth centres unaccompanied, and sometimes being without their partner during labour and childbirth. Similar findings were reported in the National Maternity Survey 2020 [37], which included over 4000 women who gave birth during the first wave of the COVID-19 pandemic. Eighty-one percent of women reported exclusion of birth partners from appointments, 60% reported partners were unable to attend scans and three-quarters said partners were restricted from attending in the early stages of labour and childbirth. Research drawing on the experience of maternity care provision in 32 European countries during the pandemic highlights that the actions taken to keep mothers and babies safe, which led to restricted contact between pregnant women and professionals, partners and babies, may lead to negative impacts on maternal psychosocial functioning and early parenting and child development [38]. At the time there were calls for maternity services to reverse these deviations from best practice, and as the pandemic evolved, NHS England did issue guidance for allowing pregnant women to have a support person of their choice with them at all antenatal, intrapartum and postnatal contacts [39]. The extent to which this guidance was implemented across the UK during the rest of the pandemic is unclear; but there are indications that restrictions on partner attendance in maternity services in England were inconsistent [40]. This undermines established global evidence on the importance of partner presence during labour, and at antenatal and postnatal contacts, to improve outcomes for women, new-borns and families [41, 42].

That several women in our study reported feeling anxious, and experienced deterioration in their mental health during the pandemic is perhaps unsurprising, but concerning, and is substantiated in other contemporaneous research. For example, the National Maternity Survey 2020 [37] found more women reported feeling anxious during pregnancy (22% compared to 13% pre-pandemic), fewer women felt involved in decisions about their pregnancy care (54% down from 70%) and more women reported postnatal anxiety (39%) and depression (22%) compared to pre-pandemic rates of 29% and 16% respectively. Other surveys reported similarly high levels of perinatal anxiety and depression [43]. Our findings indicate the importance of identifying women at risk of mental ill health during pregnancy and the postnatal period and reducing deviation from the recommendations of professional standard setting bodies [44].

The substantial and sometimes rapid modifications to maternity services during the pandemic meant some aspects of women-centred care were compromised and new norms emerged. A national survey of UK maternity services found diverse service modifications to antenatal, postnatal and intrapartum services [9]. Some of these changes, such as a reduction in the number of antenatal contacts and postnatal appointments, use of remote consultations and reduced options for place of birth run contrary to principles of women-centred care [19]. The pressures of the pandemic meant services reverted to a model of care that met the needs of the institution and the health professionals, rather than women, their partners and families. This was not unique to maternity services [45]. In our study, women recognised the challenges faced by the NHS during the pandemic and overall, most women were positive about the care received in antenatal services, at the time of birth and immediately after. Although most women did receive statutory visits from the health visitor, this did not feel sufficient and caused concern, especially first-time mothers who may need more reassurance and support. By the time of the third wave interviews, women regarded the lack of continuity of carer as the norm, but they clearly missed the drop-in weighing clinics for reassurance and valued more contact with the health visitor. Replacement of in-person health visitor contact with telephone calls also impacted negatively on women’s experience and in line with other research, women found these remote contacts disappointing, less intimate, shorter and difficult to participate in [12]. The reduction of in-person visits may reflect staff shortages due to redeployment or absence during the pandemic, but there are now clear mandates for reinstatement of health visiting services and evaluation of virtual contacts to determine their effects and impact on family outcomes [46, 47].

Our study provided examples of women steering their own way through unknown circumstances during the pandemic. While there were clearly concerns about navigating the COVID-19 recovery period with a new baby and anxiety about leaving babies with others, for some this period was also associated with benefits of increased interaction among babies and more social contact and peer support for parents. The government rapid response to the impact of the pandemic on early child development acknowledged that changed access to education and care has impacted on children’s social, emotional and behavioural development both positively and negatively depending on how families experienced the pandemic [48]. As maternity services start to reconfigure in the COVID-19 recovery period, it will be important to consider if and how to resume the aspects of postnatal care that women and partners value such as face-to-face drop-in clinics, more contact with health visitors in between scheduled contacts and flexibility in the type of consultation with the GP.

### Strengths and limitations

The main strength of the study is that we followed the same cohort of women longitudinally, across three time points from pregnancy to the postnatal period. To our knowledge, this is the only longitudinal qualitative interview study with women about this topic to date internationally. Interviewing the same women during each wave of the research allowed the interviewers to build up a rapport leading to more trusting conversations than perhaps would have happened using more traditional ‘one time’ interviews. The longitudinal design also allowed us to access women’s experiences over time to understand how and why those experiences changed [49] in response to the rapidly shifting healthcare environment during the pandemic. The timing of the study is also a strength; we began recruiting women in May 2020, just after the UK had experienced the first peak of the pandemic. We continued to interview women during the second and third lockdowns (Sept 2020-Feb 2021), and up until the Government’s roadmap for relaxing lockdowns, acceleration of vaccination and the post-COVID recovery period (June 2021 onwards). Women were able to discuss their experiences as the pandemic was unfolding, which may have minimised the likelihood of recall bias. Our sampling yielded a mix of primarily White British and Pakistani Heritage women, of different parity, but only two women were non-English language speaking. It is possible that the use of the Index of Multiple Deprivation [50] is of limited value in Bradford, where many families (particularly those of Pakistani Heritage) choose to live within the same locality regardless of socio-economic or employment status. Therefore, we may have missed the opportunity to include voices of women from lower socio-economic status, and the findings should be interpreted with this in mind. In addition, access to and use of maternity services among Urdu and other non-English language speaking women may vary from the experiences presented here.

### Implications for practice and policy

The findings from our longitudinal qualitative study have informed local and national services about women’s experiences of care during the pandemic. Midwives and other maternity staff in the local Trust where the research took place requested reports of the findings [51–53] after each wave of the research because they found them helpful in gauging the quality of care provided during periods of intense service modification. The findings informed local decisions about how to best direct resources to reducing the short- and longer-term impact of COVID-19 on women during pregnancy and postnatally. On a national level, the findings, together with other related research in the UK, have been synthesised through the work of the Parent-Infant Covid Organisational Academic Learning (PIVOT-AL) collaborative, comprising maternal and child health researchers exploring the effects of COVID-19 on health and care. Evidence generated by this group has influenced policy guidance and reports sent to chief midwives and members of parliament.

Our research shows that women and families bore the brunt of the resulting reduction in care quality brought about by service modifications. The restrictions on birth partners during antenatal contacts, labour and in childbirth and the reduced contact with health visitors postnatally, were the biggest sources of anxiety and stress for women. These restrictions also led to women feeling that they lacked access to the emotional and social support they needed. As maternity services start to reconfigure after the pandemic, policy makers should consider these as essential components of maternity care that should not be compromised in the event of future pandemics or health system shocks.

The reports of poor mental health in most women during pregnancy, and of high levels of social anxiety in their young children are of potential concern to the longer-term developmental outcomes of children born during the pandemic. Continued follow-up of these children to understand the longer-term impacts and enable the implementation of additional support for any issues identified is warranted. Table 3 summarises the key recommendations arising from this study.

**Table 3:** Key recommendations from the research

There is a need for **a balance between infection prevention and control measures and the mental wellbeing of women and socio-emotional development of children.** Having a partner at scans, during labour *and* birth, and on the postnatal wards is important to women; as is the opportunity for social support for mothers and socialisation of young children
**Face to face appointments,** not phone calls, are important to women. There is a risk that phone calls result in a lack of needed support and a failure to identify and address issues of concern
Create **a single source** of consistent, accurate, up to date and understandable **information on COVID-19 risks** for midwives, health visitors and women. This is essential to avoid confusion and misinformation
Create **a resource** women can use to find information on managing after birth, and where to seek **additional help for breastfeeding, perinatal mental health, parenting.** This is essential whilst there are less face-to-face visits
Actively **encourage services to creatively adapt,** as they did in the pandemic, to allow for optimum care within each service

Some of these recommendations could be implemented fairly quickly by services, for example ensuring that guidance for any future pandemics state clearly the need for a partner to always be present with women at critical points across their maternity journey, and providing a resource for women to find local information and services to help them manage after birth. Other recommendations, such as creating a single source of information on COVID-19 (or any future epidemic/pandemic) for women, midwives and health visitors; reinstating face to face appointments; ensuring babies and young children are able to socialise; and encouraging services to adapt to allow for optimum care, will require systems-level change from the relevant professional societies and Government endorsement. Such change takes time but as each of these recommendations are pragmatic and clear, should be achievable. To achieve the final recommendation of longer-term follow-up of children born during the pandemic, this will need to become an on-going priority for research funders and researchers alike.

## Conclusion

We found examples of excellent care and support from maternity services during the COVID-19 pandemic and maternity staff should be commended for striving to provide the best possible care in challenging circumstances. This longitudinal study allowed women to reflect, at three critical time points, on what was important to them and what they valued during their maternity journey. The accounts presented here should be used to help local decision makers restore and re-instate aspects of care that are important to women including enabling women to have a partner of choice with them at all stages of their maternity care, regular contact with health visitors and choice about remote or in-person contact from maternity staff.

## Supplementary Information

**Additional file 1.** 

**Additional file 2.** 

## Data Availability

The datasets used and/or analysed during the current study are available from the corresponding author on reasonable request.

## Abbreviations

BiB: Born in Bradford cohort study
BiB4All: Born in Bradford data linkage cohort study
COVID-19: Coronavirus Disease first recognised in 2019
JCVI: Joint Committee on Vaccination and Immunisation
NHS: National Health Service
PPE: Personal protective equipment
RCOG: Royal College of Obstetricians and Gynaecologists
WHO: World Health Organization
