# Supplementary material for: Being pregnant and becoming a parent during the COVID-19 pandemic: a longitudinal qualitative study with women in the Born in Bradford COVID-19 research study
Source: BMC Pregnancy Childbirth. 2023 Jul 4;23:494. doi: 10.1186/s12884-023-05774-4 (PMC10320984; doi:10.1186/s12884-023-05774-4)
Supplement: Supplementary file 1 — Additional file 1. [file 12884_2023_5774_MOESM1_ESM.docx]

**Additional file 1.** Topic guides

**WAVE 1**

### **Experiences of pregnancy during the COVID-19 pandemic**

First of all, I’d like to ask you some general questions about your life before and during lockdown, and specifically about your experiences of being pregnant during the COVID-19 pandemic.

1. It would be great to have some **introduction to your life when lockdown went into effect** (March 2020).

| Could you tell me about where you live (location, type of home)?  What do you do (for work/school)? | Who is in your household (does this include your partner? Children?)  Has this changed since lockdown (March)? |
| --- | --- |
| How many children do you have (is this your first child/pregnancy)? | Are you originally from [Bradford]? (If not, where are you from originally? For how long have you lived in [Bradford]?) |
| When are you expecting your baby *or* when was your baby born? | (If baby is born, remind them that the focus today is pregnancy; next time we will ask about life with the new baby) |

1. Now could you tell me generally about your **experience of being pregnant during** the time of the pandemic?

- What *have been/were* your main concerns for yourself during this time? Why?
- What *have you enjoyed/did you enjoy* about being pregnant at this time? Why?
- What *have been/were* your main concerns about the baby during this time? Why?
- What have you been least concerned about for the baby? Why?

1. How **safe *do/did* you feel** being pregnant during the COVID-19 pandemic?

- *Do/did* you have concerns about catching COVID-19 while pregnant?
- How *do/did* others feel about your safety during this time? (How does that make you feel?)
- *Have you done/did you do* anything differently during your pregnancy to keep yourself safe during the COVID-19 pandemic? (e.g. avoiding public transport, avoiding people with COVID-19 symptoms, working from home, social distancing, personal protection like handwashing)
- What could be done to make you feel safer?

1. During your pregnancy, *have/did* you or anyone else in your household fallen ill with **symptoms of coronavirus** or been tested positive for the virus? Who?

- How did you feel about this, being pregnant?
- How did you deal with this as a household?
- What did you do to keep yourself safe? (e.g self- and/or family isolation)

1. How *have you felt/did you feel* during pregnancy in terms of your **general health and fitness**?

- Do you have any concerns about your health and fitness? What are these?
- What were you doing for physical activity before lockdown in March? How did that change with the lockdown measures?
- Would you say that COVID-19 has affected your level of health and fitness?

1. How *is/was* your general **mood**?

- Have you felt down or depressed during pregnancy?
- How has COVID-19 affected your general mood?
- Have you sought any support or help when you have felt down or depressed? What/where?
- How helpful was the support or care you received?

1. Have you felt/did you feel **anxious** about being pregnant during the pandemic?

- What has made you feel anxious?
- What have you done to ease the feeling of anxiety?
- How has COVID-19 affected your level of worry or anxiety?
- What could be done for pregnant women to help relieve anxiety at this time?

1. What *were you/have you been* **most** **worried or anxious** about during your pregnancy? (e.g labour/childbirth, money/financial issues, preparation for the new baby, being a good parent)

- What have you been most worried or anxious about in the last week?
- How has this affected you?
- What have you done about this? Have you sought any support or help?
- How helpful was the support you received?

### **Plans for childbirth**

Next, I’d like to ask about your plans for giving birth and how you *feel/felt* about childbirth during the pandemic.

1. **How *did you feel/are you are feeling* about birth during the pandemic?**

- Thinking back to early this year, before the lockdown (February), how did you envision your childbirth experience?
- Have those plans changed at all? How so?
- Where *did you/do you now intend to* give birth?
- Why did you choose this option?
- How *do/did* you feel about any changes?

1. How *do/did* you feel about **giving birth in a hospital setting** during the pandemic?

- What *concerns/concerned* you most about this?
- *Do/did* you worry about exposure to COVID-19 while in hospital?
- *Do/did* you worry about your new baby being exposed to COVID-19 in the hospital? (If not, why?)
- *Are/were* others concerned for you? How *does/did* that make you feel?
- Have you sought any help or support to relieve those concerns? (e.g. self-help, previous experience, midwife, partner, other)?
- How helpful was the support you received?

### **Access to and use of antenatal and pregnancy services**

Next, I’d like to ask you a few questions about your use of antenatal and other services during pregnancy. *[If applicable: Remember that we are focussing now on your time before the birth. We will speak again in a few months to talk about your experiences after the birth.]*

1. **Please tell me generally about your experience of accessing midwifery led services during the time of the pandemic.**

- *Have you been attending/did you attend* midwifery appointments? Why, why not?
- *Have you experienced/did you experience* any difficulties accessing midwife appointments during this time?
- *What have been/were* the positive aspects of accessing care at this time? Why?
- Do you feel you *have received/did receive* enough support from the midwife during COVID-19?

1. Can you tell me **where do you usually go** for your midwife appointment?

- What mode of interaction have you had with the midwife (e.g. phone call, video or online call, face to face)
- What has been your experience of this mode of interaction? Positive and negative points?
- Do you see the same person every time? How do you feel about this?
- How would you describe your relationship with the midwife?
- Is there someone else you would rather have seen? Who, why?

1. Would you have **liked anything to be different** about your midwife appointments?

- More or fewer appointments? Why?
- At what point would you have liked more appointments? Why?
- Would you have preferred a different length of appointment? Why?
- Would you have preferred another method of interaction (e.g. phone, video, face to face)? Why?
- Is anyone allowed to attend midwife appointments with you?
- Who usually attends antenatal care with you? Would you have preferred someone else?

1. During the pandemic, **how satisfied** have you been with the midwifery care you have received?

- What have been the best and worst aspects of the care? Why?
- Have you received enough information about how COVID-19 affects pregnancy and birth?
- What else would you like to have known about?
- What could be done to make it easier for women to attend midwifery appointments during the pandemic?
- How does your experience of antenatal care during COVID-19 compare with before the pandemic? (or with previous pregnancies)
- Have you seen/did you see a **doctor** at all during your pregnancy?
- [If yes:] How frequently, and for what reason(s)? *Have you seen/did you see* the same person each time?

1. *Are you attending/did you attend* any **parenting classes** to help prepare you for the birth and looking after your baby?

- If yes, where *do/did* you attend parenting classes? Who delivers the classes?
- What mode of interaction (e.g. face to face, video or phone)?
- *Have you been/were you* able to attend regularly? Why, why not?
- Do you feel you have received enough support from parenting classes during COVID-19?
- If you *are not attending/did not attend*, why not? What stopped you attending? What could have been done to enable you to attend?

### **Relationship with partner and family**

I’d like to know more about your relationships with your partner, friends and family during your pregnancy, especially how things may have changed during the pandemic. [If needed: Could you confirm who makes up your household?/Do you have a partner?]

1. **Please tell me who you *regard/regarded* as important to you during your pregnancy?**

- Why *are/were* they particularly important to you during pregnancy?
- What impact has the outbreak and lockdown had on these relationships?
- How *has this affected/did this affect* you during pregnancy?

1. How **involved *has your partner been/was your partner* when you first knew you were pregnant**? How involved *have they been/were they* throughout your pregnancy?

- Would they have liked to be more involved? In what way/why?
- Have they been able to attend appointments with you?
- How would you describe your relationship with your partner throughout your pregnancy? Did the lockdown affect this?
- Has COVID-19 affected your partner’s involvement in your pregnancy in any other ways?
- How do you think they have felt about this? How has this affected them? And your relationship?

1. Tell me a bit about your **family network** – who *you can/did you* count on most during your pregnancy?

- How easy is it usually for you to reach out to these people if you need help?
- What has changed in terms of your contact with these people during the COVID-19 pandemic?
- How *has this affected/did this affect* you during pregnancy?

1. Tell me a bit about your **social network** – who do you see or speak to regularly?

- How easy is it for you to reach out to these people if you need help?
- What has changed in terms of your contact with these people during the COVID-19 pandemic?
- How has this affected you?
- *Have you been/were you* able to make new friends or contacts while pregnant? Who have you met?
- How easy or difficult has it been to make new friends or contacts during the COVID-19 outbreak? Why?

### **General impact of COVID-19 on services**

1. **Thinking about your pregnancy overall, what would you say about how your local midwife and antenatal care services have responded to the COVID-19 crisis?**

- What has been done well? What has not been done well?
- What else can be done to help pregnant women and their partners cope during the outbreak?
- Are there any positive changes to services that should continue after the pandemic? What are these and why?
- Are there any negative impacts that need to be addressed? What are these and why?

### **Wrap up**

We have come to the end of the interview. I don’t have any more questions for you, but do you have anything else you would like to say about what we have discussed today?

**WAVE 2**

Before we start, can I check some details about your current circumstances and whether anything has changed since we last spoke.

| Could you tell me about where you live (location, type of home)?  **Has this changed since we last spoke?**  What do you do (for work/school)?  **Has this changed?** | Who is in your household (does this include your partner? Children?)  **Has this changed since we last spoke?** |
| --- | --- |
| How many children do you have (is this your first child/pregnancy)? | Are you originally from [Bradford]? (If not, where are you from originally? For how long have you lived in [Bradford]?) |
| When was your baby born? | (Remind them that the focus today is about life with the new baby) |

### **Experiences of childbirth during the COVID-19 pandemic**

First of all, I’d like to ask you some general questions about your experiences of childbirth and how you felt about giving birth during the pandemic.

1. Please tell me about your **birth experience?**

- Did your plans for the birth change at all? How so?
- How did you feel about the changes?
- Where did you give birth?
- Why did you choose this option?
- Who was with you at the birth? How did you decide who would join you, and when? Was this what you wanted/expected?
- How did COVID-19 rules/restrictions impact on your birth experience (e.g. navigating the rules, ability to change birth partner, childcare issues)

2. How did you feel about **giving birth in a hospital (or other setting)** during the pandemic?

- What worries or concerns did you have?
- Did you worry about exposure to COVID-19 while in hospital (or other setting)?
- Did you worry about your new baby being exposed to COVID-19 in the hospital (or other setting)?
- Were others concerned for you? How did that make you feel?
- Who did you turn to for help or support to relieve those concerns? (e.g. self-help, previous experience, midwife, partner, other)?
- How helpful was the support you received?

### **Immediate postnatal period during the COVID-19 pandemic**

Now some questions about your postnatal experience, and looking after yourself and your baby during the COVID-19 pandemic.

1. Could you tell me generally about your **experience of looking after a newborn baby during** the pandemic?

- How is your relationship with your baby?
- How has the pandemic and lockdown influenced your relationship with your baby?
- What have been your main concerns for your baby during this time? Why?
- What have been your main concerns about yourself during this time? Why?
- What have you enjoyed about being mother to a newborn at this time? Have there been any benefits to having a newborn during the pandemic/lockdown?
- What have you least enjoyed about having a newborn during the pandemic/lockdown?

1. How **safe do you feel being mother to a newborn** during the COVID-19 pandemic/most recent lockdown?

- Do you have concerns about catching COVID-19?
- Do you have concerns about your baby catching COVID-19?
- Have you received any information about how COVID-19 affects new mothers/newborns?
- Do you have concerns about your baby’s lack of exposure to others during lockdown?
- How do others in your family feel about your and your baby’s safety during this time?
- Have you done anything differently to keep yourself and your baby safe during the COVID-19 pandemic? (e.g. avoiding public transport, avoiding people with COVID-19 symptoms, social distancing, personal protection like handwashing)
- What could be done to make you feel safer?

1. During the last few months after giving birth, **did you or anyone else in your household fall ill with symptoms of coronavirus** or been tested positive for the virus? Who?

- How did you feel about this, being at home with a newborn?
- How did you deal with this as a household?
- What did you do to keep yourself and baby safe? (e.g self- and/or family isolation)

1. How have you felt since giving birth, in terms of your **general health and fitness**?

- Do you have any concerns about your health and fitness? What are these?
- How has your physical activity changed since we last spoke?
- How has being a new mum impacted on your health and fitness?
- Would you say that COVID-19 has affected your level of health and fitness?

1. How is your general **mood at the moment**?

- Have you felt down or depressed since having your baby?
- Have you felt lonely or alone?
- How has COVID-19/most recent lockdown affected your general mood?
- Have you sought any support or help when you have felt down or depressed? What/where?
- How helpful was the support or care you received?

1. What have you been **most** **worried or anxious** about since having your baby? (e.g being alone, lack of social contact, money/financial issues, caring for the new baby, being a good parent, lockdown and returning to normal life)

- What have you been most worried or anxious about in the last week?
- How has this affected you?
- What have you done about this? Have you sought any support or help?
- How helpful was the support you received?
- What could be done for postnatal women to help relieve anxiety at this time?

### **Access to and use of postnatal care and related services**

Next, I’d like to ask you a few questions about your experience of postnatal and other services since having your baby.

1. Please tell me **generally about your experience of postnatal care** during the time of the pandemic?

- Did the midwife or health visitor visit you at home after you had your baby? (why not?)
- What was your experience of postnatal home visits during COVID-19 restrictions? What worked well, what didn’t work well?
- Have you experienced any difficulties accessing postnatal care during this time?
- Are there services for your baby that you have not been able to access because of COVID-19 (e.g. weighing/measuring baby, developmental assessment, immunisations)? What impact has this had on you, your baby?
- Are there any services for you that you have not been able to access? (e.g. breastfeeding support, weight loss support, health check following birth). What impact has this had on you?
- Do you feel you have received enough support from midwives/health visitors during COVID-19?
- What have been the positive aspects of accessing postnatal care at this time? What could be better/different?

1. Can you tell me about any **alternatives to face to face visits** that you have been offered for postnatal care during the pandemic? (e.g. video call, phone call)

- What mode of interaction have you experienced with the midwife/health visitor?
- What has been your experience of this mode of interaction? Positive and negative points?
- Do you see the same person every time? How do you feel about this?
- How would you describe your relationship with the midwife/health visitor?
- Is there someone else you would rather have seen? Who, why?

1. Would you have **liked anything to be different** about the postnatal care you received?

- More or fewer visits/contacts? Why?
- At what point would you have liked more visits/contacts? Why?
- Would you have preferred a different length of visit/contact? Why?
- Would you have preferred another method of interaction (e.g. phone, video, face to face)? Why?
- Was your partner allowed to be with you during midwife/health visitor visits/contact? (Why not?)
- What could be done to make it easier for women to see the midwife/health visitor during the pandemic?

1. During the pandemic, **how satisfied have you been with the postnatal care** you have received from the midwife/health visitor?

- What have been the best and worst aspects of the care? Why?
- Have you received enough information about how COVID-19 affects you and your baby?
- What else would you like to have known about?
- How does your experience of postnatal care during COVID-19 compare with before the pandemic? (or with previous pregnancies)
- Have you seen a **doctor** at all since having your baby?
- [If yes:] what was your experience? Positive and negative points?

1. Please tell me about any **community-based groups you attend** with your baby? (e.g. parent and baby groups, breastfeeding support, social or other groups)

- Where do you attend the groups? Who facilitates the groups?
- What is the mode of interaction (e.g. face to face, video or phone)? What has been your experience of this mode of interaction? Which mode works best for you, why?
- Have you been able to join groups regularly? Why, why not?
- If not, how has this affected you? Your baby?
- If you are not attending any groups why not? What has prevented you from attending? What could have been done to enable you to attend?
- Do you feel you have missed out on anything because of not attending, or not attending face to face during the pandemic?

### **Relationship with partner and family**

I’d like to know more about your relationships with your partner, friends and family since having your baby, especially how things may have changed during the pandemic. [If needed: Could you confirm who makes up your household?/Do you have a partner?]

1. Please tell me **about your home life** since you had your baby?

- How have you and your partner adjusted to having a newborn at home?
- How have any siblings adjusted?
- What impact has COVID-19 and the January lockdown restrictions had on this adjustment?

1. **Who has been important to you** since having your baby?

- Why have they been particularly important to you?
- What impact has the outbreak and lockdown had on these relationships?
- How has this affected you in the period since giving birth?

1. How **involved has your partner been since you had your baby**?

- e.g. feeding, playing, changing nappies, helping around the house
- Would they have liked to be more involved? In what way/why?
- Have they been able to attend parent-baby groups or community-based classes with your baby?
- How would you describe your relationship with your partner since you had your baby? How has the lockdown affected this?
- Has COVID-19 affected your partner’s involvement with the baby/ you in any other ways?
- How do you think they have felt about this? How has this affected them? And your relationship?

1. Tell me a bit about your **family network** – who you can count on most since having your baby?

- What kind of support do they provide to you (and the baby)?
- How has the pandemic and lockdown affected your contact with family members?
- Have you been able to see family members in person since having your baby?
- If not, how has this affected you? And your baby?
- How was the December holiday period for you? Who were you able to see?
- How did this affect you? What would you have preferred to do/ who would you have liked to see? Why?

1. Tell me a bit about your **social network** – who do you see or speak to regularly since having your baby?

- How easy is it for you to reach out to these people if you need help?
- What has changed in terms of your social network during the COVID-19 pandemic?
- How has this affected you?
- How easy or difficult has it been to make new friends or contacts during the COVID-19 outbreak? Why?

### **General impact of COVID-19 on services**

Part of this study is about feedback to maternity and community services on what they are doing well during the pandemic and what they can improve. We value your views on this.

1. Thinking about the period since you gave birth, overall, what would you say about how your local midwife and postnatal care services have responded to the COVID-19 crisis?

- What has been done well? What has not been done well?
- What else could the services have done to help you, your partner and your baby during the pandemic?
- Are there any positive changes to services that should continue after the pandemic? What are these and why?
- Are there any negative impacts that need to be addressed? What are these and why?

### **Wrap up**

We have come to the end of the interview. I don’t have any more questions for you, but do you have anything else you would like to say about what we have discussed today?

**WAVE 3**

Before we start, can I check some details about your current circumstances and whether anything has changed since we last spoke.

| Could you tell me about where you live (location, type of home)?  **Has this changed since we last spoke?**  What do you do (for work/school)?  **Has this changed?** | Who is in your household (does this include your partner? Children?)  **Has this changed since we last spoke?** |
| --- | --- |
| How many children do you have (is this your first child/pregnancy)? | Are you originally from [Bradford]? (If not, where are you from originally? For how long have you lived in [Bradford]?) |
| When was your baby born? | (Remind them that the focus today is about the last 6 months) |

### **Experiences of caring for your baby**

First of all, I’d like to ask you some questions about looking after yourself and your baby during the last 6 months.

1. Could you tell me generally about your **experience of looking after your baby during the last 6 months** of the pandemic?

- How is your relationship with your baby?
- How has the pandemic and lockdown influenced your relationship with your baby?
- What have been your main concerns for your baby during this time? Why?
- What have been your main concerns about yourself during this time? Why?
- What have you enjoyed about being mother to a baby at this time? Have there been any benefits to having a baby during the pandemic/lockdown?
- What have you least enjoyed about having a baby during the pandemic/lockdown?

1. How **safe do you feel being mother to a baby** during the last 6 months of the COVID-19 pandemic, as restrictions have started to ease?

- Do you have concerns about catching COVID-19?
- Do you have concerns about your baby catching COVID-19?
- What has your baby’s exposure to others been like in the last 6 months? In the last 3 months?
- How/do you think this has affected them in terms of their development? (Could you share any examples?)
- Do you have concerns about your baby’s exposure to others now restrictions have been lifted?
- How do you feel about seeing other people in your family or social groups now restrictions have eased?
- How do others in your family feel about your and your baby’s safety during this time?
- Have you done anything differently to keep yourself and your baby safe during this time? (e.g. avoiding public transport, avoiding people with COVID-19 symptoms, social distancing, personal protection like handwashing)
- What could be done to make you feel safer / less anxious during the reopening period?

1. During the last 6 months, **did you or anyone else in your household fall ill with symptoms of coronavirus** or been tested positive for the virus? Who?

- How did you feel about this, being at home with a baby?
- How did you deal with this as a household?
- What did you do to keep yourself and baby safe? (e.g self- and/or family isolation)

1. How have you felt in the last 6 months, in terms of your **general health and fitness**?

- How has your health and fitness changed across the different stages of lockdown over the last 6 months? How has this affected you?
- What do you do in terms of physical activity now that restrictions have eased?
- Do you have any concerns about your health and fitness? What are these?
- How has being a new mum impacted on your health and fitness?

1. How is your general **mood at the moment**?

- Have you felt down or depressed in the last 6 months?
- Have you felt lonely or alone?
- How has the easing of COVID-19 restrictions affected your general mood?
- Have you sought any support or help when you have felt down or depressed? What/where?
- How helpful was the support or care you received?

1. What have you been **most** **worried or anxious** about in the last 6 months? (e.g being alone, lack of social contact, money/financial issues, caring for the new baby, being a good parent, lockdown and returning to normal life)

- What have you been most worried or anxious about in the last week?
- How has this affected you?
- What have you done about this? Have you sought any support or help?
- How helpful was the support you received?
- What could be done for postnatal women to help relieve anxiety at this time?

### **Access to and use of health care services including postnatal care**

Next I’d like to ask you a few questions about your experience of using health care services in the last 6 months, including community-based postnatal care.

1. Please tell me **about your experience of community-based postnatal care** (provided by midwife and/or health visitors) in the last 6 months ?

- Have you had any mother and baby assessments since we last spoke? (why not?)
- Has your baby been offered the usual course of vaccinations? If so, what was your experience of that/those appointment(s)?
- Did you have, or have you got an appointment for, the one-year assessment? (why not?)
- What was your experience of these assessments - what worked well and less well? How does this compare to previous experiences (if any older children, or during the pandemic)?
- What was the mode of interaction for these assessments (e.g. face to face/videocall/phone)?
- What do you think about this mode of interaction? Positive and negative points?
- Do you see the same person every time? How do you feel about this?
- How would you describe your relationship with the midwife/health visitor?
- Was your partner allowed to accompany you to the assessments?
- Is there any way these assessments could have been improved?

1. Please tell me **about your experience of other community support services (e.g. run by children’s centres, family services or local clinics)** in the last 6 months?

- Have you used any support services in the last 6 months? (e.g or breastfeeding, nutrition, sleep support, parenting, mental health, women’s health)
- What was your experience of these services – what worked well and less well?
- What was the mode of interaction for these services (e.g. face to face/video call/phone)?
- What do you think about this mode of interaction? Positive and negative points?
- Was your partner allowed to accompany you to any appointments/these services?
- Is there any way these services could have been improved?

1. What would you say **generally about the community services** you have used during the pandemic in the last 6 months?

- What have been the best and worst aspects?
- Are there services for you or your baby that you have not been able to access because of COVID-19 (e.g. weighing/measuring baby, developmental assessment, immunisations, own health check, weight loss support etc)
- What impact has this had on you, your baby?
- Do you feel you have received enough information and support from midwives/health visitors during COVID-19?
- How satisfied have you been with the community postnatal care you have received?
- What has changed since the easing of restrictions? Good and bad aspects?

1. Please tell me about any **community-based groups you attend with your baby since we last spoke**? (e.g. parent and baby groups, social or other groups)

- What has your experience been of these groups – what have you enjoyed, not enjoyed?
- What was the mode of interaction (e.g. face to face, video or phone)? What has been your experience of this mode of interaction? Which mode works best for you, why?
- Have you been able to join groups regularly? Why, why not? Has your partner joined?
- If not, how has this affected you? Your baby?
- If you are not attending any groups why not? What has prevented you from attending? What could have been done to enable you to attend?
- Do you feel you have missed out on anything because of not attending, or not attending face to face during the pandemic?
- How have these groups changed since the easing of restrictions? Good and bad aspects?

### **Relationship with partner and family**

I’d like to know more about your relationships with your partner, friends and family in the last 6 months, especially how things may have changed with the easing of COVID-19 restrictions. [If needed: Could you confirm who makes up your household?/Do you have a partner?]

1. **Who has been important to you** over the last 6 months?

- Why have they been particularly important to you?
- What impact has the easing of restrictions had on these relationships?
- How has this affected you and your baby?

1. How **involved has your partner been in the last 6 months**?

- (e.g. feeding, playing, changing nappies, helping around the house)
- Would they have liked to be more involved? In what way/why?
- Have they been able to attend parent-baby groups or community-based classes with your baby?
- How would you describe your relationship with your partner recently? How has this changed with the recent easing of restrictions?
- How do you think they have felt about this? How has this affected them?

1. Tell me a bit about your **family network** – and how things have changed in the last 6 months?

- How has the pandemic and lockdown affected your contact with family members?
- Has anyone in your family relied on you for care in the last 6 months? How has this impacted you?
- How has this changed with the easing of restrictions?
- How do you feel about having more contact with family members?
- How has this affected you? And your baby?

1. Tell me a bit about your **social network** – who have you seen or spoken to regularly in the last 6 months?

- How easy is it for you to reach out to these people if you need help?
- What has changed since the easing of restrictions?
- How has this affected you? And your baby?
- How easy or difficult has it been to make new friends or contacts during the last 6 months? Why?
- How have you felt about resuming social activities and the possibility of meeting new parents, or making new friends?

### **General impact of COVID-19 on services**

Part of this study is about feedback to maternity and community services on what they are doing well during the pandemic and what they can improve. We value your views on this.

1. Thinking about the period since you gave birth, overall, what would you say about how your local midwife and postnatal care services have responded to the COVID-19 crisis?

- How has your experience of services changed (for better or worse) throughout the pandemic?
- What has been done well? What has not been done well?
- What else could the services have done to help you, your partner and your baby during the pandemic?
- Are there any positive changes to services that should continue after the pandemic? What are these and why?
- Are there any negative impacts that need to be addressed? What are these and why?
- What has been the most difficult challenge during your time as a new mum in a pandemic, and how did / could services have supported you? What has been the best aspect of being a mum during this time, and what are you looking forward to the most now?

### **Wrap up**

We have come to the end of the interview. I don’t have any more questions for you, but do you have anything else you would like to say about what we have discussed today?
