# Supplementary material for: Being pregnant and becoming a parent during the COVID-19 pandemic: a longitudinal qualitative study with women in the Born in Bradford COVID-19 research study
Source: BMC Pregnancy Childbirth. 2023 Jul 4;23:494. doi: 10.1186/s12884-023-05774-4 (PMC10320984; doi:10.1186/s12884-023-05774-4)
Supplement: Supplementary file 2 — Additional file 2. [file 12884_2023_5774_MOESM2_ESM.docx]

**Additional file 2.** Wave 1 framework

| **Category** | **Code** | **Sub-codes** |
| --- | --- | --- |
| Introduction to Life/Background | Ethnicity |  |
|  | Profession |  |
|  | Partner's profession |  |
|  | Living arrangement |  |
|  | Household composition |  |
|  | Number of children |  |
|  | First pregnancy |  |
|  | Caring responsibilities |  |
|  | Current location |  |
|  | Originally from |  |
|  | Bradford residency time |  |
|  | Baby expected/born |  |
|  | Other |  |
| Experience of COVID-19 (not about pregnancy) | Work/employment | Working situation before/after pandemic began |
|  |  | Partner's working situation before/after pandemic began |
|  | Lifestyle | Socialising |
|  |  | Finances |
|  |  | Holidays |
|  |  | Lack of plans/cancellations |
|  | Actions to manage risk of infection | Cleaning (house/surfaces/shopping) |
|  |  | PPE |
|  |  | Self-isolation |
|  |  | Avoiding public transport |
|  | Thoughts on managing risk | Government response (national) |
|  |  | Restrictions (local) |
|  | Comparing stages of pandemic (early vs late) |  |
|  | Other |  |

| Experience of pregnancy | Main concerns during pregnancy | Lack of information |
| --- | --- | --- |
|  |  | Concern for partner contracting COVID-19 |
|  |  | Concern for self contracting COVID-19 |
|  |  | Concern for baby contracting COVID-19 |
|  | Actions to manage risks of COVID-19 re: pregnancy |  |
|  | Sense of safety during pregnancy |  |
|  | Fitness | Self-described fitness/activity level |
|  |  | Activity levels decreased |
|  |  | Activity levels maintained |
|  | Physical health | Pre-existing condition |
|  | Mood/mental health | Self-described mental health |
|  |  | Low mood |
|  |  | Anxiety |
|  |  | No change in mood/mental health |
|  |  | Good mood |
|  | Comparison with previous pregnancies |  |
|  | Other |  |
| Plans for childbirth | Giving birth alone |  |
|  | COVID-19 risk in hospital |  |
|  | Birthing partner(s) |  |
|  | Birthing mode |  |
|  | Plans for birth/changing plans |  |
|  | General/non-COVID-19 reflections |  |
|  | Previous birth experiences/comparisons |  |
|  | Other |  |
| Experience of birth | Birth experience (actual) |  |
|  | Post-birth |  |
|  | Other |  |
| Antenatal services | General experience accessing services | COVID-19 risks accessing services |
|  |  | Wishes for antenatal services |
|  |  | General satisfaction with services |
|  |  | Accessing other medical services |
|  |  | Previous antenatal services experiences/comparison |
|  |  | Attending alone |
|  |  | Partner experience |
|  | Midwife appointments | Number of appointments |
|  |  | Mode of appointment delivery |
|  | Midwife relationships | Continuity of care |
|  |  | Description of relationship with midwife(s) |
|  | Parenting classes | Virtual vs in-person availability of classes |
|  |  | Attendance |
|  |  | Significance |
|  |  | Awareness of availability |
|  | Other |  |
| Relationships and support networks | Experience of support during pregnancy | Important people |
|  |  | Type/method of support |
|  |  | Impact of pandemic on support |
|  | Relationship with partner | Partner (non) involvement |
|  |  | Change in relationship |
|  | Other family network |  |
|  | Friend/social network | Maintaining friend networks |
|  |  | Creating new friendships |
|  | Comparison with previous pregnancies |  |
|  | Other |  |
| Other general | Other negative impacts |  |
|  | Other positives and silver linings |  |
|  | Other wishes |  |
